# Supplementary material for: Molecular mechanism of Arp2/3 complex inhibition by Arpin
Source: Nat Commun. 2022 Feb 2;13:628. doi: 10.1038/s41467-022-28112-2 (PMC8810855; doi:10.1038/s41467-022-28112-2)
Supplement: Supplementary file 1 — Supplementary Information [file 41467_2022_28112_MOESM1_ESM.pdf]

Molecular mechanism of Arp2/3 complex inhibition by Arpin

Fregoso *et al*.

Supplementary Information

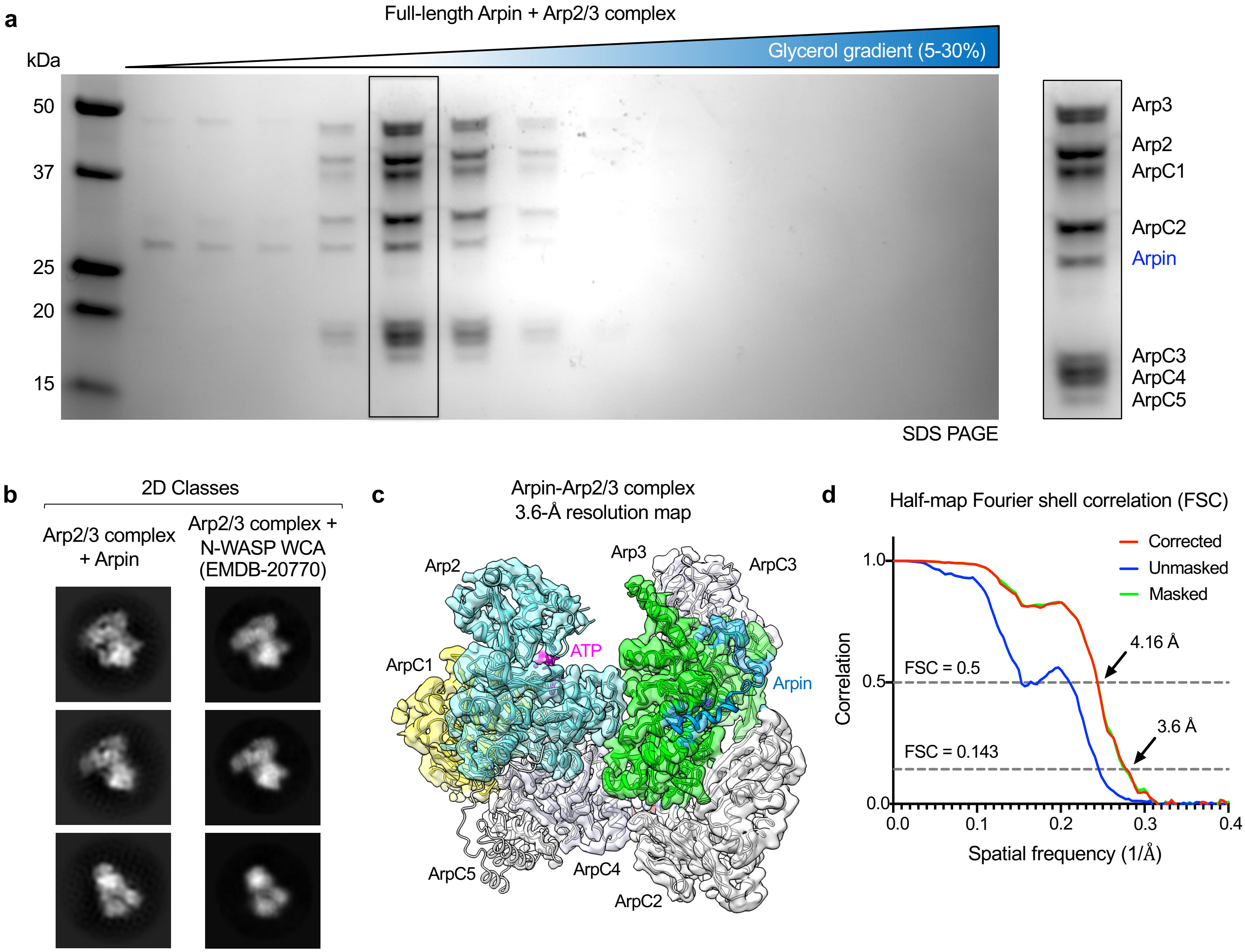

Supplementary Fig. 1. Sample preparation and cryo-EM analysis of full-length Arpin bound to Arp2/3 complex. a, SDS-PAGE analysis of glycerol gradient centrifugation fractions of bovine Arp2/3 complex (8.6 μM) mixed with a 2-fold molar excess of full-length human Arpin. The inset highlights the peak fraction used for cryo-EM analysis, containing the seven subunits of Arp2/3 complex and Arpin. Excess Arpin appears in the first three fractions. In order to troubleshoot cryo-EM grids, this gradient was repeated with reproducible results. **b.** Comparison of 2D classes of Arp2/3 complex with bound full-length Arpin (left) and N-WASP WCA (right). Density corresponding to the globular domain of Arpin was not observed, suggesting that it is flexible and thus unresolved in the structure. **c**, 3D reconstruction of full-length Arpin bound to Arp2/3 complex resulting from a final subset of 68,318 particles (out of 4,485,086 picked particles). The map is semi-transparent, and ribbon diagrams of Arp2/3 complex subunits are also shown. Arp2/3 complex subunits are labeled and colored as figure 1 of the main text. **d**, FSC analysis defines a map resolution of 3.6-Å at FSC = 0.143.

Supplementary Figure 2

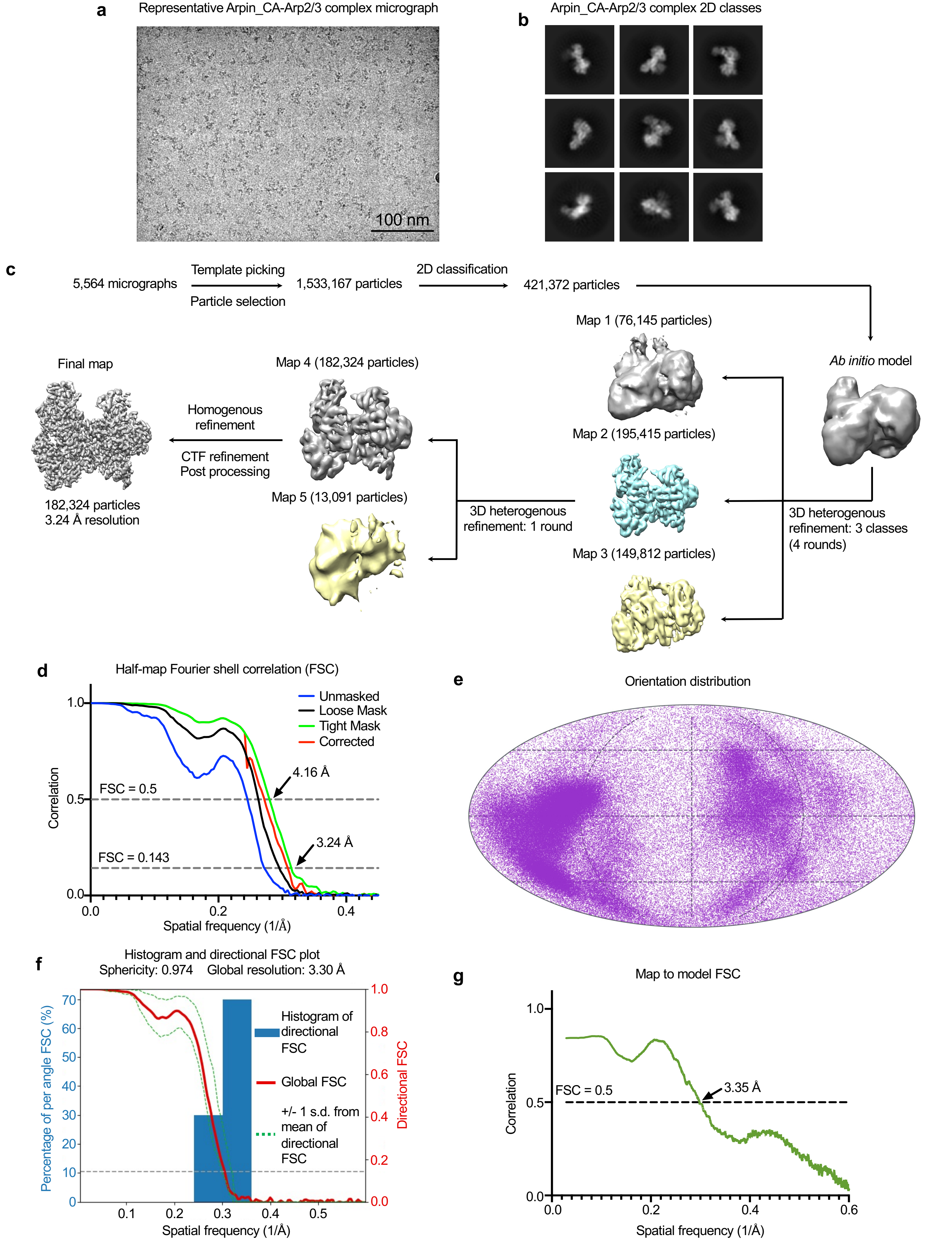

Supplementary Fig. 2. Cryo-EM image processing and workflow. a, Representative micrograph of Arpin_CA-Arp2/3 complex (scale bar: 100 nm). Troubleshooting for optimum ice thickness and particle distribution allowed for reproducible results of this representative micrograph. **b.** cryoSPARC-generated 2D class averages of Arpin_CA bound to Arp2/3 complex, showing secondary structure information. **c.** Cryo-EM workflow (see Methods). A total of 5,564 micrographs were used to template pick 1,533,167 particles using 2D classes from the complex of full-length Arpin bound Arp2/3 complex in cryoSPARC. A subset of 421,372 particles were selected from 2D classification and used to generate an *ab initio* model. Four rounds of heterogenous classification using the *ab initio* model as reference yielded a map with 195,415 particles (Map 2) that had clear density for Arpin_CA. This was followed by one round of heterogenous refinement using a dummy class to remove junk particles. The resulting 182,324 particles map (Map 4) was used for iterative rounds of homogenous refinement and local CTF refinement in cryoSPARC to yield the final map. Post processing of this map was performed with deepEMhancer. **d**, FSC analysis defines the resolution of the map as 3.24-Å at FSC = 0.143. **e**, Orientation distribution of particles from the final reconstruction as determined with the program cryoEF. The final map has a calculated efficiency (E_{od}) of 0.78. **f**, 3D Fourier shell correlation calculated using the 3D-FSC server. The map shows high sphericity (0.974 out of 1) and a global resolution of 3.3-Å. **g**, Map to model FSC determined with the program Phenix.

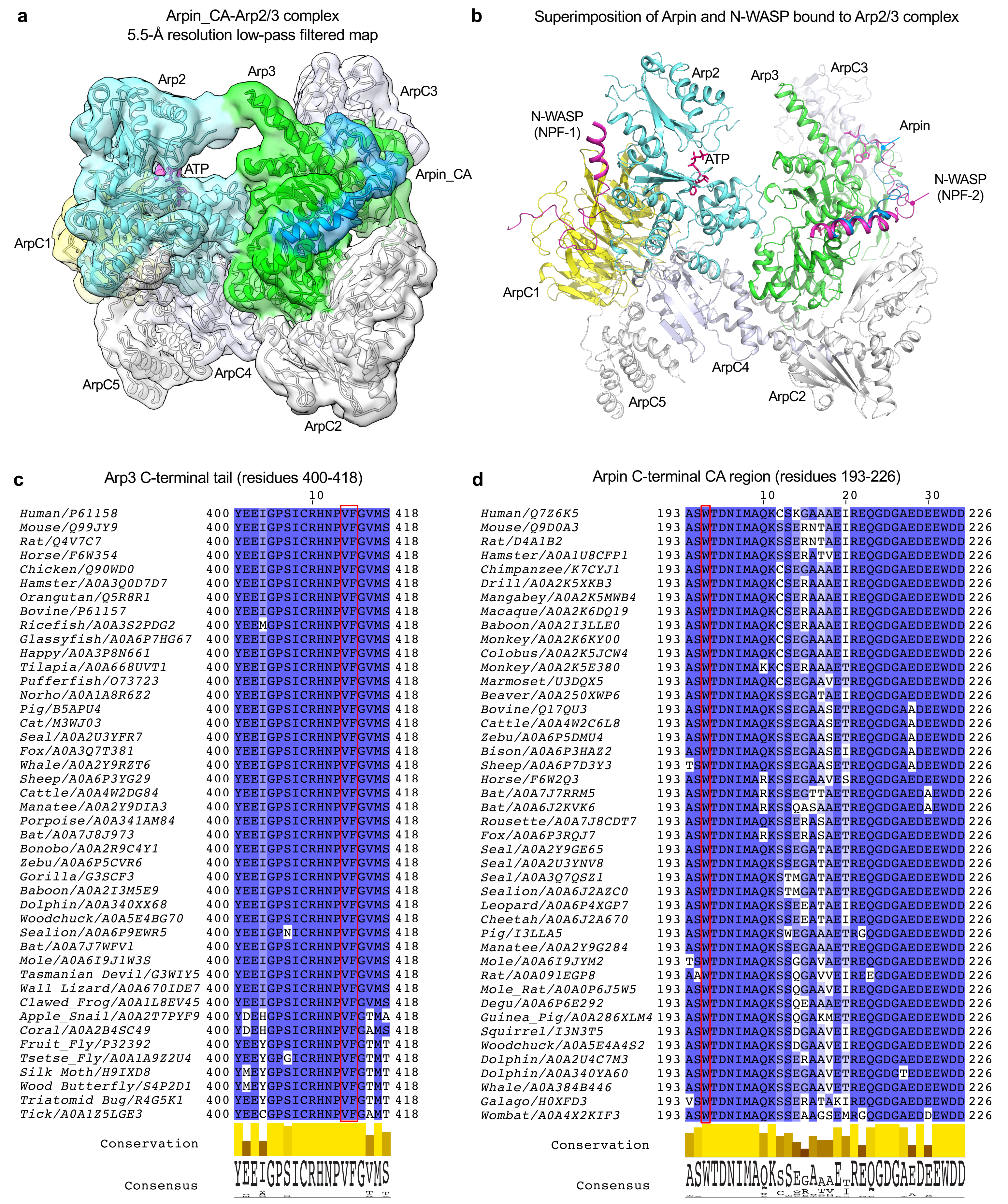
a
Arpin_CA-Arp2/3 complex
5.5-Å resolution low-pass filtered map
Arp2
Arp3
ArpC3
ATP
Arpin_CA
ArpC1
ArpC5
ArpC4
ArpC2
b
Superimposition of Arpin and N-WASP bound to Arp2/3 complex
Arp2
Arp3
ArpC3
N-WASP (NPF-1)
ATP
Arpin
N-WASP (NPF-2)
ArpC1
ArpC4
ArpC5
ArpC2
c
Arp3 C-terminal tail (residues 400-418)
10
Human/P61158 400 YEEIGPSICRHNPVFGVMS 418
Mouse/Q99JY9 400 YEEIGPSICRHNPVFGVMS 418
Rat/Q4V7C7 400 YEEIGPSICRHNPVFGVMS 418
Horse/F6W354 400 YEEIGPSICRHNPVFGVMS 418
Chicken/Q90WD0 400 YEEIGPSICRHNPVFGVMS 418
Hamster/A0A3Q0D7D7 400 YEEIGPSICRHNPVFGVMS 418
Orangutan/Q5R8R1 400 YEEIGPSICRHNPVFGVMS 418
Bovine/P61157 400 YEEIGPSICRHNPVFGVMS 418
Ricefish/A0A3S2PDG2 400 YEEMGPSICRHNPVFGVMS 418
Glassyfish/A0A6P7HG67 400 YEEIGPSICRHNPVFGVMS 418
Happy/A0A3P8N661 400 YEEIGPSICRHNPVFGVMS 418
Tilapia/A0A668UVT1 400 YEEIGPSICRHNPVFGVMS 418
Pufferfish/O73723 400 YEEIGPSICRHNPVFGVMS 418
Norho/A0A1A8R6Z2 400 YEEIGPSICRHNPVFGVMS 418
Pig/B5APU4 400 YEEIGPSICRHNPVFGVMS 418
Cat/M3WJ03 400 YEEIGPSICRHNPVFGVMS 418
Seal/A0A2U3YFR7 400 YEEIGPSICRHNPVFGVMS 418
Fox/A0A3Q7T381 400 YEEIGPSICRHNPVFGVMS 418
Whale/A0A2Y9RZT6 400 YEEIGPSICRHNPVFGVMS 418
Sheep/A0A6P3YG29 400 YEEIGPSICRHNPVFGVMS 418
Cattle/A0A4W2DG84 400 YEEIGPSICRHNPVFGVMS 418
Manatee/A0A2Y9DIA3 400 YEEIGPSICRHNPVFGVMS 418
Porpoise/A0A341AM84 400 YEEIGPSICRHNPVFGVMS 418
Bat/A0A7J8J973 400 YEEIGPSICRHNPVFGVMS 418
Bonobo/A0A2R9C4Y1 400 YEEIGPSICRHNPVFGVMS 418
Zebu/A0A6P5CVR6 400 YEEIGPSICRHNPVFGVMS 418
Gorilla/G3SCF3 400 YEEIGPSICRHNPVFGVMS 418
Baboon/A0A2I3M5E9 400 YEEIGPSICRHNPVFGVMS 418
Dolphin/A0A340XX68 400 YEEIGPSICRHNPVFGVMS 418
Woodchuck/A0A5E4BG70 400 YEEIGPSICRHNPVFGVMS 418
Sealion/A0A6P9EWR5 400 YEEIGPNICRHNPVFGVMS 418
Bat/A0A7J7WFV1 400 YEEIGPSICRHNPVFGVMS 418
Mole/A0A6I9J1W3S 400 YEEIGPSICRHNPVFGVMS 418
Tasmanian Devil/G3WIY5 400 YEEIGPSICRHNPVFGVMS 418
Wall Lizard/A0A670IDE7 400 YEEIGPSICRHNPVFGVMS 418
Clawed Frog/A0A1L8EV45 400 YEEIGPSICRHNPVFGVMS 418
Apple Snail/A0A2T7PYF9 400 YDEHGPSICRHNPVFGTMA 418
Coral/A0A2B4SC49 400 YDEHGPSICRHNPVFGAMS 418
Fruit Fly/P32392 400 YEEYGPSICRHNPVFGTMT 418
Tsetse Fly/A0A1A9Z2U4 400 YEEYGPGICRHNPVFGTMT 418
Silk Moth/H9IXD8 400 YMEYGPSICRHNPVFGTMT 418
Wood Butterfly/S4P2D1 400 YMEYGPSICRHNPVFGTMT 418
Triatomid Bug/R4G5K1 400 YEEYGPSICRHNPVFGTMT 418
Tick/A0A1Z5LGE3 400 YEECGPSICRHNPVFGAMT 418
Conservation
Consensus
YEEIGPSICRHNPVFGVMS
d
Arpin C-terminal CA region (residues 193-226)
10 20 30
Human/Q7Z6K5 193 ASWTDNIMAQKCSKGAAAEIREQGDGAEDEEWDD 226
Mouse/Q9D0A3 193 ASWTDNIMAQKSSERNTAEIREQGDGAEDEEWDD 226
Rat/D4A1B2 193 ASWTDNIMAQKSSERNTAEIREQGDGAEDEEWDD 226
Hamster/A0A1U8CFP1 193 ASWTDNIMAQKSSERATVEIREQGDGAEDEEWDD 226
Chimpanzee/K7CYJ1 193 ASWTDNIMAQKCSEGAAAEIREQGDGAEDEEWDD 226
Drill/A0A2K5XKB3 193 ASWTDNIMAQKCSERAAAEIREQGDGAEDEEWDD 226
Mangabey/A0A2K5MWB4 193 ASWTDNIMAQKCSERAAAEIREQGDGAEDEEWDD 226
Macaque/A0A2K6DQ19 193 ASWTDNIMAQKCSERAAAEIREQGDGAEDEEWDD 226
Baboon/A0A2I3LLE0 193 ASWTDNIMAQKCSERAAAEIREQGDGAEDEEWDD 226
Monkey/A0A2K6KY00 193 ASWTDNIMAQKCSEGAAAEIREQGDGAEDEEWDD 226
Colobus/A0A2K5JCW4 193 ASWTDNIMAQKCSEGAAAEIREQGDGAEDEEWDD 226
Monkey/A0A2K5E380 193 ASWTDNIMAKKCSERAAAETREQGDGAEDEEWDD 226
Marmoset/U3DQX5 193 ASWTDNIMAQKCSEGAAVETREQGDGAEDEEWDD 226
Beaver/A0A250XWP6 193 ASWTDNIMAQKSSEGATAEIREQGDGAEDEEWDD 226
Bovine/Q17QU3 193 ASWTDNIMAQKSSEGAASETREQGDGAADEEWDD 226
Cattle/A0A4W2C6L8 193 ASWTDNIMAQKSSEGAASETREQGDGAADEEWDD 226
Zebu/A0A6P5DMU4 193 ASWTDNIMAQKSSEGAASETREQGDGAADEEWDD 226
Bison/A0A6P3HAZ2 193 ASWTDNIMAQKSSEGAASEIREQGDGAADEEWDD 226
Sheep/A0A6P7D3Y3 193 TSWTDNIMAQKSSEGAASETREQGDGAADEEWDD 226
Horse/F6W2Q3 193 ASWTDNIMARKSSEGAAVESREQGDGAEDEEWDD 226
Bat/A0A7J7RRM5 193 ASWTDNIMARKSSEGTTAETREQGDGAEDAEWDD 226
Bat/A0A6J2KVK6 193 ASWTDNIMARKSSQASAAETREQGDGAEDAEWDD 226
Rousette/A0A7J8CDT7 193 ASWTDNIMAQKSSERASAETREQGDGAEDEEWDD 226
Fox/A0A6P3RQJ7 193 ASWTDNIMARKSSERASAETREQGDGAEDEEWDD 226
Seal/A0A2Y9GE65 193 ASWTDNIMAQKSSEGATAETREQGDGAEDEEWDD 226
Seal/A0A2U3YNV8 193 ASWTDNIMAQKSSEGATAETREQGDGAEDEEWDD 226
Seal/A0A3Q7QSZ1 193 ASWTDNIMAQKSTMGATAETREQGDGAEDEEWDD 226
Sealion/A0A6J2AZC0 193 ASWTDNIMAQKSTMGATAETREQGDGAEDEEWDD 226
Leopard/A0A6P4XGP7 193 ASWTDNIMAQKSSEEATAETREQGDGAEDEEWDD 226
Cheetah/A0A6J2A670 193 ASWTDNIMAQKSSEEATAEIREQGDGAEDEEWDD 226
Pig/I3LLA5 193 ASWTDNIMAQKSWEGAAAETRGQGDGAEDEEWDD 226
Manatee/A0A2Y9G284 193 ASWTDNIMAQKSSEGAAAETREQGDGAEDEEWDD 226
Mole/A0A6I9JYM2 193 TSWTDNIMAQKSSGGAVAETREQGDGAEDEEWDD 226
Rat/A0A091EGP8 193 AAWTDNIMAQKSSQGAVVEIREEGDGAEDEEWDD 226
Mole_Rat/A0A0P6J5W5 193 ASWTDNIMAQKSSQGAAVEIREQGDGAEDEEWDD 226
Degu/A0A6P6E292 193 ASWTDNIMAQKSSQEAAAETREQGDGAEDEEWDD 226
Guinea_Pig/A0A286XLM4 193 ASWTDNIMAQKSSQGAKMETREQGDGAEDEEWDD 226
Squirrel/I3N3T5 193 ASWTDNIMAQKSSDGAAVEIREQGDGAEDEEWDD 226
Woodchuck/A0A5E4A4S2 193 ASWTDNIMAQKSSDGAAVEIREQGDGAEDEEWDD 226
Dolphin/A0A2U4C7M3 193 ASWTDNIMAQKSSERAAVETREQGDGAEDEEWDD 226
Dolphin/A0A340YA60 193 ASWTDNIMAQKSSEGAAVETREQGDGTEDEEWDD 226
Whale/A0A384B446 193 ASWTDNIMAQKSSEGAAVETREQGDGAEDEEWDD 226
Galago/H0XFD3 193 VSWTDNIMAQKSSERATAKIREQGDGAEDEEWDD 226
Wombat/A0A4X2KIF3 193 ASWTDNIMAQKSSEAAGSEMRGQGDGAEDDEWDD 226
Conservation
Consensus
ASWTDNIMAQKSSEGAAAETREQGDGAEDEEWDD

Supplementary Fig. 3. Binding of Arpin CA to Arp3. a, Cryo-EM map (including a ribbon diagram of Arp2/3 complex subunits) low-pass filtered to 5.5-Å resolution of Arpin CA (marine blue) bound to Arp2/3 complex (subunits labeled and colored as in figure 2c of the main text). **b**, Ribbon diagram of the structure of Arpin CA (marine blue) bound to Arp2/3 complex, showing a superimposition of the two binding sites of N-WASP CA (magenta, PDB code 6UHC). **c**,**d**, Sequence alignment of the C-termini of Arp3 (**b**) and Arpin (**c**) highlighting Arp3 residues V413 and F414 and Arpin residue W195 (of the C-helix), which participate in hydrophobic interactions that help stabilize the inactive conformation of Arp2/3 complex. Blue shading of decreasing intensity (dark blue to light blue) indicates strictly to highly conserved amino acids. UniProt accession codes are given with the name of each sequence.

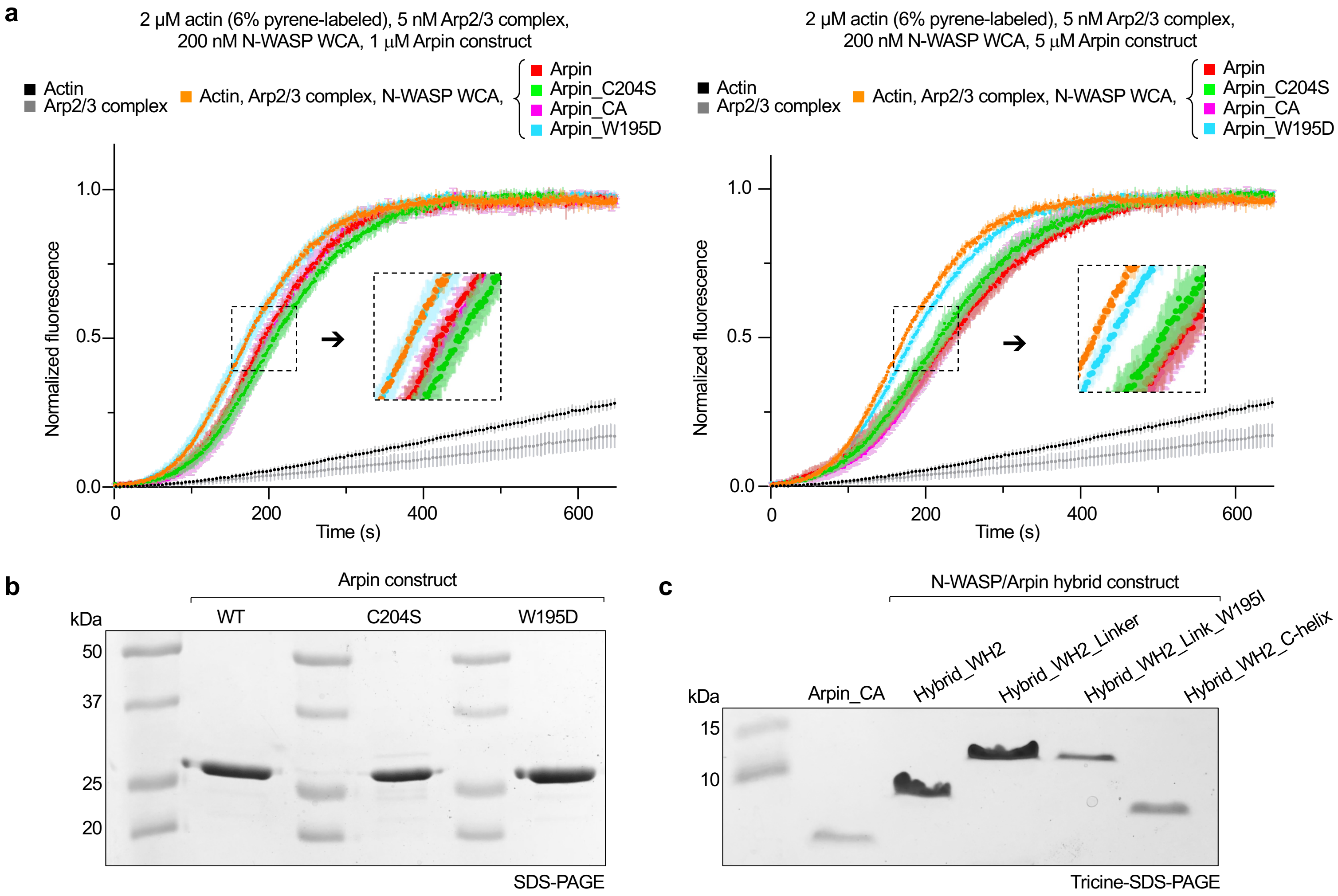

Supplementary Fig. 4. Inhibition of actin polymerization by Arpin constructs. **a**, Time-course of actin polymerization by Arp2/3 complex activated by N-WASP WCA, with and without the indicated Arpin constructs (at two different concentrations). The concentrations of the proteins are indicated. Data are shown as the average curve from three independent experiments with s.d. error bars in lighter color. Insets show a zoom of experimental curves that overlap. **b**,**c**, SDS-PAGE analysis of the of Arpin (**b**) and hybrid N-WASP-Arpin (**c**) constructs described in figure 4a. Protein expression and purification was optimized for all Arpin and N-WASP/Arpin hybrid constructs.

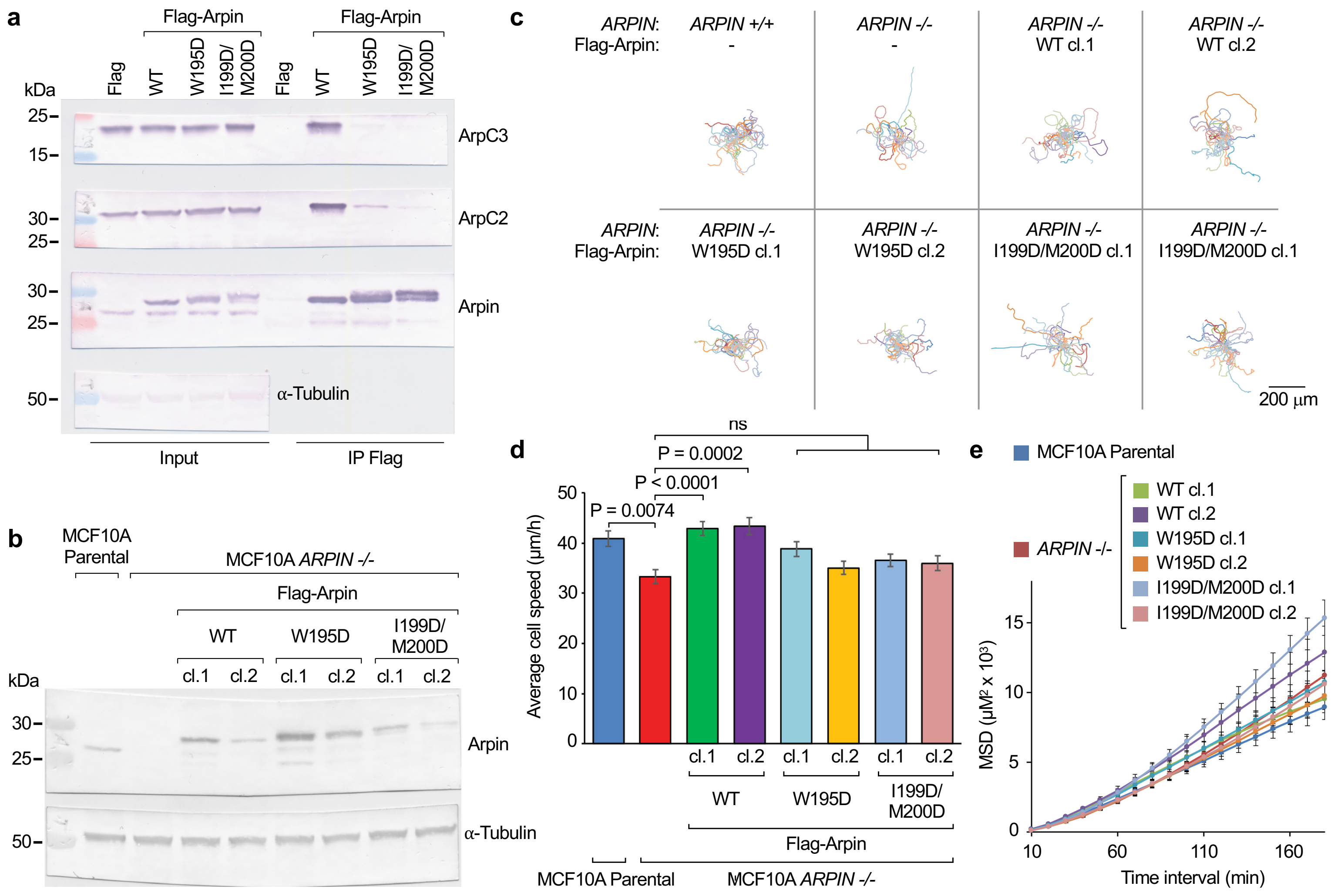

Supplementary Fig. 5. Analysis of Arpin C-helix mutants in cells. a,**b**, Raw Western blots for panels a and b of figure 5 in main text. Immunoprecipitations (a) were extensively repeated during experimental optimization, and western blot of MCF10A clones stably expressing the specified Arpin construct (b) were repeated for optimum detection. **c**, Single cell trajectories tracked for 7h using ImageJ. Representative trajectories of 25 cells (out of a total of 67-74 cell) is shown for each Arpin expression condition and cell line. **d**, Average cell speed for each Arpin expression condition and cell line. The statistical significance of the measurements was calculated using a Kruskal-Wallis test (exact P-values are listed in the figure). Source Data are provided as a Source Data file. **e**, Mean square displacement as a function of time for MCF10A cells and for each Arpin expression condition and cell line. N = 74 cells for MCF10A parental, ARPIN -/- and WT cl.1, N = 72 cells for WT cl.2, N = 75 cells for W195D cl.1, N = 69 cells for W195D cl.2, N = 76 cells for I199D/M200D cl.1, and N = 67 cells for I199D/M200D cl.2. Results are expressed as means and standard error of the mean.

Table S1 | Primers used in this study

Construct name	Primer sequence
Arpin_C204S (forward)	gcccaaaagtcttcgaagggg
Arpin_C204S (reverse)	catgatgttgtctgtccag
Arpin_W195D (forward)	ggaaagacaggggcatccgacacagacaacatcatggcccaaaag
Arpin_W195D (reverse)	cttttgggccatgatgttgtctgtgtcggatgcccctgtctttcc
Arpin_CA (forward)	ttaactagtagctggacagacaatatcatggcgcag
Arpin_CA (reverse)	ttagcggccgcttagcagccggatccccttcctgcagt
Hybrid_WH2_Linker (forward)	agaagacttcgaggaagacgaagaatgggac
Hybrid_WH2_Linker (reverse)	cgtcgtcctcgtcatctccctgttcacggat
Hybrid_WH2_Linker_W195I (forward)	cgcccgcgcctacctctagcatcacagacaatatcatggcgc
Hybrid_WH2_Linker_W195 (reverse)	gcgccatgatattgtctgtgatgctagaggtaggcgcgggcg
Hybrid_WH2_C-helix (forward)	atgattctgatcatcagtttttgcttggatcc
Hybrid_WH2_C-helix (reverse1)	cattcttcgtcttccgccccgtcttcatcttcatctgaggaatgaatggc
Hybrid_WH2_C-helix_(reverse2)	ttcgaattctcagtcgtcccattcttcgtcttccgcccc
Site-directed mutagenesis	
Arpin_W195D (forward)	gaaagacaggggcatccgacacagacaacatcatgg
Arpin_W195D (reverse)	ccatgatgttgtctgtgtcggatgcccctgtctttc
Arpin_I199D/M200D (forward)	gcatcctggacagacaacgacgacgcccaaaagtgttcgaag
Arpin_I199D/M200D (reverse)	cttcgaacacttttgggcgtcgtcgttgtctgtccaggatgc
