# Supplementary material for: Molecular mechanism of Arp2/3 complex inhibition by Arpin
Source: Nat Commun. 2022 Feb 2;13:628. doi: 10.1038/s41467-022-28112-2 (PMC8810855; doi:10.1038/s41467-022-28112-2)
Supplement: Supplementary file 3 — Reporting Summary [file 41467_2022_28112_MOESM3_ESM.pdf]

nature portfolio

Corresponding author(s): Roberto Dominguez

Last updated by author(s): 2021/12/23

Reporting Summary

Nature Portfolio wishes to improve the reproducibility of the work that we publish. This form provides structure for consistency and transparency in reporting. For further information on Nature Portfolio policies, see our Editorial Policies and the Editorial Policy Checklist.

Statistics

For all statistical analyses, confirm that the following items are present in the figure legend, table legend, main text, or Methods section.

n/a	Confirmed	
☐	☒	The exact sample size (*n*) for each experimental group/condition, given as a discrete number and unit of measurement
☐	☒	A statement on whether measurements were taken from distinct samples or whether the same sample was measured repeatedly
☐	☒	The statistical test(s) used AND whether they are one- or two-sided<br>*Only common tests should be described solely by name; describe more complex techniques in the Methods section.*
☒	☐	A description of all covariates tested
☒	☐	A description of any assumptions or corrections, such as tests of normality and adjustment for multiple comparisons
☐	☒	A full description of the statistical parameters including central tendency (e.g. means) or other basic estimates (e.g. regression coefficient) AND variation (e.g. standard deviation) or associated estimates of uncertainty (e.g. confidence intervals)
☐	☒	For null hypothesis testing, the test statistic (e.g. *F*, *t*, *r*) with confidence intervals, effect sizes, degrees of freedom and *P* value noted<br>*Give P values as exact values whenever suitable.*
☒	☐	For Bayesian analysis, information on the choice of priors and Markov chain Monte Carlo settings
☒	☐	For hierarchical and complex designs, identification of the appropriate level for tests and full reporting of outcomes
☒	☐	Estimates of effect sizes (e.g. Cohen's *d*, Pearson's *r*), indicating how they were calculated

Our web collection on statistics for biologists contains articles on many of the points above.

Software and code

Policy information about availability of computer code

Data collection	Latitude (Gatan) was used for cryo-EM data collection.<br>Data collection of cell migration are described in Methods section.
Data analysis	Data processing for cryo-EM and single particle analysis, MotionCorr2, Relion-3.0.8, CTFFIND-4.1.13, and cryoSPARC-2.12.4. Post processing of the Arpin CA-Arp2/3 complex map was performed with deepEMhancer, and the orientation distribution of particles from the final reconstruction was determined using cryoEF-v1.1.0. The 3DFSC server was used to calculate the 3D-FSC of the final map. The program Coot-0.9.3 was used for model building, and the program Phenix-1.19.2 was used for model refinement. Maps for figures were low-pass filtered using the program e2proc3d within the EMAN2 suite, and figures were prepared using the programs Chimera-1.15, ChimeraX-1.2.4, and PyMOL-2.5.2. For ITC analyses, the program Origin-7.0 was used to analyze the raw binding isotherms. Domain diagrams were made in PowerPoint-16.53, multiple sequence alignment was performed in Jalview-2.11.1.4 using ClustalW-1.2.2 and edited in Adobe Illustrator-25.4.1. For pyrene-actin polymerization assay, Prism-7.0 was used for statistical analysis and graphing. Single cell trajectories were tracked with ImageJ-1.53k14 and migration parameters, directional autocorrelation, mean square displacement, average cell speed, and single cell trajectories were calculated and plotted using the DiPer suite. The statistical analysis of migration persistence, measured as the movement autocorrelation over time, was performed using the program R-4.0.4.

For manuscripts utilizing custom algorithms or software that are central to the research but not yet described in published literature, software must be made available to editors and reviewers. We strongly encourage code deposition in a community repository (e.g. GitHub). See the Nature Portfolio guidelines for submitting code & software for further information.

Data

Policy information about availability of data

All manuscripts must include a data availability statement. This statement should provide the following information, where applicable:

- Accession codes, unique identifiers, or web links for publicly available datasets
- A description of any restrictions on data availability
- For clinical datasets or third party data, please ensure that the statement adheres to our policy

Cryo-EM maps and models were deposited in the Electron Microscopy Data Bank (EMDB-22416 [https://www.ebi.ac.uk/pdbe/entry/emdb/EMD-22416] and the atomic coordinates were deposited in the Protein Data Bank (accession code: 7JPN [https://doi.org/10.2210/pdb7JPN/pdb]). PDB codes 6YW7 [http://doi.org/10.2210/pdb6YW7/pdb] and 6UHC [http://doi.org/10.2210/pdb6UHC/pdb] were used for Figure 1.

Field-specific reporting

Please select the one below that is the best fit for your research. If you are not sure, read the appropriate sections before making your selection.

- [x] Life sciences
- [ ] Behavioural & social sciences
- [ ] Ecological, evolutionary & environmental sciences

For a reference copy of the document with all sections, see nature.com/documents/nr-reporting-summary-flat.pdf

Life sciences study design

All studies must disclose on these points even when the disclosure is negative.

Sample size	Sample sizes for pyrene-actin polymerization assays of N = 3 was chosen based on previous experiences with this specific type of experiment and commonly used sample size in the field (Madasu et al 2015, Drazic et al 2018, and Zimmet et al 2020). No predetermination of sample size was performed for cell migration experiments. The standard in the field is about 50 cells. This allows to capture significant differences on migration parameters studied here, speed, MSD, migration persistence. We repeated the experiment 3 times to track enough single cells, from 67 to 74 cells, depending on the condition, with N specified in the appropriate figure legends.
Data exclusions	All data corresponding to a technically sound experiment (which did not fail for an obvious reason) are shown. No data were excluded from analysis.
Replication	All biochemical and cell biological experiments in this study have been reproduced using the same experimental set-up (proteins, buffers, cell extracts, and independent biological experiments) with similar results. For Western blots, representative results are shown. The number of independent replications is stated in the Figure Legends.
Randomization	Stable clones expressing either Arpin WT or mutants W195D or I199D/M200D were derived from the MCF10A ARPIN -/- cell line. Two clones expressing these variants close to endogenous Arpin levels were randomly selected and further studied. Samples (cells) were allocated to each group according to the genome modification they harbored.
Blinding	There was no blinding for the analysis of cell migration. The person who performed the videomicroscopy acquisition also analyzed the resulting movies. At the time, there was only a single person in the lab trained for videomicroscopy and able to analyze the resulting movies.

Reporting for specific materials, systems and methods

We require information from authors about some types of materials, experimental systems and methods used in many studies. Here, indicate whether each material, system or method listed is relevant to your study. If you are not sure if a list item applies to your research, read the appropriate section before selecting a response.

Materials & experimental systems

n/a	Involved in the study	
[ ]	[x]	Antibodies
[ ]	[x]	Eukaryotic cell lines
[x]	[ ]	Palaeontology and archaeology
[x]	[ ]	Animals and other organisms
[x]	[ ]	Human research participants
[x]	[ ]	Clinical data
[x]	[ ]	Dual use research of concern

Methods

n/a	Involved in the study	
[x]	[ ]	ChIP-seq
[x]	[ ]	Flow cytometry
[x]	[ ]	MRI-based neuroimaging

Antibodies

Antibodies used	Polyclonal antibodies targeting Arpin were obtained in rabbit (Agro-Bio) against purified human full length Arpin, and then purified by

affinity purification on a HiTrap NHS-activated HP column (GE Healthcare) coupled to the immunogen. ArpC2 polyclonal rabbit antibody was from Millipore (Cat#07-227), and ArpC3 polyclonal rabbit antibody (Cat#ABN176) and and Tubulin monoclonal mouse antibody (Cat#CP06, clone DM1A) were from Sigma-Aldrich.

Validation

The affinity-purified Arpin antibodies were validated by Westen blots (Fig.3 and extended data Fig.S6 of Dang et al, 2013, Fig.4 of Lomakina BJC 2016, Fig 1 and Fig S11 of Molinie Cell Res 2019, and Fig.4 of Simanov IJMS 2021), immunoprecipitation, and immunofluorescence (Extended Data Fig.S5 of Dang et al, 2013) using parental MCF10A cells and ARPIN KO derivatives as a negative control. ArpC2 polyclonal rabbit antibody (Millipore, Cat#07-227) was validated by Western Blotting in A431 cell lysate: Western Blotting Analysis: 1 µg/mL of this antibody detected p34-Arc/ARPC2 in A431 cell lysate. ArpC3 polyclonal rabbit antibody (Sigma-Aldrich, Cat#ABN17) was validated by Western Blotting in human spleen tissue lysate: Western Blot Analysis: 0.5 µg/mL of this antibody detected ARPC3 in 10 µg of human spleen tissue lysate). Tubulin monoclonal mouse antibody (Sigma-Aldrich, Cat#CP06, clone DM1A) was validated as stated on their website: "This Anti-α-Tubulin Mouse mAb (DM1A) is validated for use in Immunoblotting, Immunofluorescence for the detection of α-Tubulin". https://www.sigmaaldrich.com/US/en/product/mm/cp06

Eukaryotic cell lines

Policy information about cell lines

Cell line source(s)

The mammary epithelial cell line MCF10A was from the collection of human breast organized at Institut Curie (Paris) by Thierry Dubois. Human embryonic kidney 293T cells were from ATCC.

Authentication

MCF10A cells were authenticated by microsatellite profiling (Institut Curie). HEK-293T are not authenticated.

Mycoplasma contamination

All cells and stable clones were routinely tested for mycoplasma and found to be negative (internal PCR assays with appropriate positive controls).

Commonly misidentified lines
(See ICLAC register)

Name any commonly misidentified cell lines used in the study and provide a rationale for their use.
